# Author’s response: Oropouche virus risk for European travellers to Cuba: an emerging public health concern

**DOI:** 10.2807/1560-7917.ES.2024.29.38.2400607

**Published:** 2024-09-19

**Authors:** Concetta Castilletti, Federico Giovanni Gobbi, on behalf of all co-authors of the original article

**Affiliations:** 1 Department of Infectious–Tropical Diseases and Microbiology, Istituto di Ricovero e Cura a Carattere Scientifico (IRCCS) Sacro Cuore Don Calabria Hospital, Negrar di Valpolicella, Verona, Italy; 2 Department of Clinical and Experimental Sciences, University of Brescia, Brescia, Italy; 3 https://www.eurosurveillance.org/content/10.2807/1560-7917.ES.2024.29.26.2400362

**Keywords:** World, vector-borne infections, zoonotic infections, viral infections, climate change, infection control, mass gatherings, public health policy, surveillance, Oropouche disease, Emerging threat, global health

**To the Editor**: Open and timely sharing of epidemiological and clinical data are the essential tool to counter epidemic emergencies such as the current Oropouche virus (OROV) disease. Therefore, we strongly agree with the views expressed by Giovanetti et al. In a recent article published in Lancet Infectious Diseases, after describing the first two cases diagnosed in Europe at the laboratory of the Department of Infectious–Tropical Diseases and Microbiology (DITM) of IRCCS Sacro Cuore Don Calabria in Negrar (Verona, Italy), our research group emphasised the importance of collaboration at every level, from international organisations to research centres and hospitals, both top-down and vice versa [1]. The experience of the past few months leads us to indicate some key points for research and public health interventions at global and local levels in the immediate future.

First, we must improve collection and distribution of data, to have an accurate and as up-to-date picture of the situation as possible. The latest epidemiological update published by Pan American Health Organization (PAHO) on 6 September 2024 reports 9,852 confirmed cases of Oropouche disease from 1 January 2024 to date; of these, 7,931 (80.5%) were recorded in Brazil and 506 (5.1%) in Cuba. And yet, the vast majority of imported cases of Oropouche disease in countries where OROV is not endemic involve travellers from Cuba: Canada (1 of 1), the United States (21 of 21), Europe (20 of 30) [2].

In addition to the increasing flow of tourists from Europe and North America to the Caribbean area, including Cuba, it should not be forgotten that starting in 2025, ca 20–30 million pilgrims are expected to converge on Rome for the Holy Year. Final pilgrims count of the last Jubilee in 2015 was 21,292,926 [3]. Many of these pilgrims will come from Latin America, where Catholicism is the prevailing religion. Therefore, it is important to strengthen international surveillance and preparedness.

Oropouche virus infection generally gives mild and self-limiting symptoms, but its overall impact is not to be overlooked. According to the above-cited PAHO update, the current OROV outbreak has resulted in one suspected and two confirmed deaths, all in Brazil, where a case of OROV-associated encephalitis has been recorded. Also in Brazil, several confirmed or suspected cases of vertical transmission of the virus were recorded: 11 fetal deaths, four congenital newborn abnormalities, three miscarriages. Further studies are needed to assess the risks to specific groups of people (e.g. children, older adults, people with comorbidities, pregnant people) [2].

Oropouche virus is mainly present in remote wilderness areas. Today, however, human movements, growing urbanisation and climate change facilitate the virus spread outside its endemic area, as has happened with West Nile virus (WNV) and dengue virus (DENV), which have become endemic in some European regions. Vector competence studies will be critical to assess the potential OROV transmission by arthropods from non-endemic areas. The main known vehicle of OROV infection is the bite of the *Culicoides paraensis* midge. However, Cuba is not among countries where *C. paraensis* presence has been detected [4]. Moreover, a 2022 review surveyed six other insect species with OROV transmission competence: five mosquito species (*Coquillettidia venezuelensis*, *Mansonia venezuelensis*, *Culex quinquefasciatus*, *Culex tarsalis*, *Aedes serratus*) and the midge *Culicoides sonorensis* [5]. In Italy, for instance, the above-mentioned mosquito species of the *Culex* and *Aedes* genera and midges of the genus *Culicoides* are widely spread.

The first imperative, in conclusion, is to learn more about this hitherto little-studied virus: epidemiology, genomic evolution and clinical impact. Collaboration and sharing of results at every level are, as always, the key to successfully containing and preventing epidemic emergencies.
